# Retraction Note: A new noninvasive method can effectively assess intracranial compliance. Letter to the Editor

**DOI:** 10.1007/s00701-025-06757-4

**Published:** 2025-12-20

**Authors:** Sérgio Brasil, Daniel Agustín Godoy

**Affiliations:** 1 Division of Neurosurgery, Department of Neurology, School of Medicine, University of São Paulo, Av. Dr. Eneas de Carvalho Aguiar 255, São Paulo, Brazil; 2 Neurointensive Care Unit, Sanatório Pasteur, Catamarca, Argentina

**Retraction Note: Acta Neurochirurgica (2023) 165:2213-2214**

10.1007/s00701-023-05644-0

The Editor-in-Chief has retracted this article because of a failure to disclose a major conflict of interest by the authors. One of the authors is on the Scientific Board of Brain4Care, this direct conflict of interest was never disclosed during the publication process. The Editor-in-Chief therefore no longer has confidence in the integrity of this article.

The author Sérgio Brasil did not explicitly state whether they agree or disagree with this retraction. The author Daniel Agustín Godoy has not responded to any correspondence about this retraction.

